# Supplementary material for: MetaQTL: a package of new computational methods for the meta-analysis of QTL mapping experiments
Source: BMC Bioinformatics. 2007 Feb 8;8:49. doi: 10.1186/1471-2105-8-49 (PMC1808479; doi:10.1186/1471-2105-8-49)
Supplement: Additional File 7 — MetaQTL Package : jar file and tutorial. This Zip archive contains both the MetaQTL JAR file and the files of the tutorial. [file 1471-2105-8-49-S7.zip › org.inra.metaqtl/doc/index-files/index-14.html]

O-Index


|  |  |  |
| --- | --- | --- |
| **Overview** \| Package \| Class \| Use \| **Tree** \| **Deprecated** \| **Index** \| **Help** | |  |
| **PREV LETTER**   **NEXT LETTER** | **FRAMES**    **NO FRAMES**     **All Classes** |  |


A B C D E F G H I K L M N O P Q R S T U V W X Y Z 

---


## **O**

**o\_mrkIdxL** - Variable in class org.metaqtl.Qtl.QTLProj: The original indice of the left flanking marker. **o\_mrkIdxR** - Variable in class org.metaqtl.Qtl.QTLProj: The original indice of the right flanking marker. **OCCURENCE** - Static variable in interface org.metaqtl.IBioLocusProperties: The key for the occurence of markers in a consensus map **ocov** - Variable in class org.metaqtl.EMResult: The observed information (just for means). **OffspringManager** - Class in org.metaqtl.util: Class Description Here **OffspringManager()** - Constructor for class org.metaqtl.util.OffspringManager: **OffspringManager.HaldaneWaddingtonProportion** - Class in org.metaqtl.util: **OffspringManager.HaldaneWaddingtonProportion()** - Constructor for class org.metaqtl.util.OffspringManager.HaldaneWaddingtonProportion: **olog** - Variable in class org.metaqtl.EMResult: The observed loglikelihood. **Ontology** - Class in org.thalia.bio.entity: **Ontology()** - Constructor for class org.thalia.bio.entity.Ontology: **ONTOLOGY** - Static variable in interface org.thalia.bio.IBioEntityTypes: **ONTOLOGY\_TERM** - Static variable in interface org.thalia.bio.IBioEntityTypes: **OntologyBean** - Class in org.thalia.bio.entity.bean: **OntologyBean()** - Constructor for class org.thalia.bio.entity.bean.OntologyBean: **OntologyBeanAdapter** - Class in org.thalia.bio.entity.adapter: **OntologyBeanAdapter()** - Constructor for class org.thalia.bio.entity.adapter.OntologyBeanAdapter: **OntologyTabFactory** - Class in org.thalia.bio.entity.factory: A factory to deal with `Ontology`. **OntologyTabFactory()** - Constructor for class org.thalia.bio.entity.factory.OntologyTabFactory: **OntologyTerm** - Class in org.thalia.bio.entity: **OntologyTerm()** - Constructor for class org.thalia.bio.entity.OntologyTerm: **OntologyTermBean** - Class in org.thalia.bio.entity.bean: **OntologyTermBean()** - Constructor for class org.thalia.bio.entity.bean.OntologyTermBean: **OntologyTermBeanAdapter** - Class in org.thalia.bio.entity.adapter: **OntologyTermBeanAdapter()** - Constructor for class org.thalia.bio.entity.adapter.OntologyTermBeanAdapter: **org.metaqtl** - package org.metaqtl: **org.metaqtl.adapter** - package org.metaqtl.adapter: **org.metaqtl.algo** - package org.metaqtl.algo: **org.metaqtl.factory** - package org.metaqtl.factory: **org.metaqtl.graph** - package org.metaqtl.graph: **org.metaqtl.main** - package org.metaqtl.main: **org.metaqtl.numrec** - package org.metaqtl.numrec: **org.metaqtl.util** - package org.metaqtl.util: **org.thalia.bio** - package org.thalia.bio: **org.thalia.bio.entity** - package org.thalia.bio.entity: **org.thalia.bio.entity.adapter** - package org.thalia.bio.entity.adapter: **org.thalia.bio.entity.bean** - package org.thalia.bio.entity.bean: **org.thalia.bio.entity.factory** - package org.thalia.bio.entity.factory: **org.thalia.bio.util** - package org.thalia.bio.util: **org.thalia.metaqtl.main** - package org.thalia.metaqtl.main

---


|  |  |  |
| --- | --- | --- |
| **Overview** \| Package \| Class \| Use \| **Tree** \| **Deprecated** \| **Index** \| **Help** | |  |
| **PREV LETTER**   **NEXT LETTER** | **FRAMES**    **NO FRAMES**     **All Classes** |  |


A B C D E F G H I K L M N O P Q R S T U V W X Y Z 

---
